# Signalling through the yeast MAPK Cell Wall Integrity pathway controls P-body assembly upon cell wall stress

**DOI:** 10.1038/s41598-019-40112-9

**Published:** 2019-02-28

**Authors:** Raúl García, Verónica Pulido, Sara Orellana-Muñoz, César Nombela, Carlos R. Vázquez de Aldana, José M. Rodríguez-Peña, Javier Arroyo

**Affiliations:** 1 Departamento de Microbiología y Parasitología, Facultad de Farmacia, Universidad Complutense de Madrid, IRYCIS, Madrid, 28040 Spain; 2 Instituto de Biología Funcional y Genómica, IBFG-CSIC. Universidad de Salamanca, Salamanca, 37007 Spain

**Keywords:** Cell signalling, Genetics

## Abstract

Post-transcriptional control of mRNA is a key event in the regulation of gene expression. From yeast to human cells, P-bodies are cytoplasmic RNA-protein aggregates that play an essential role in this process, particularly under stress conditions. In this work, we show that in the model yeast *Saccharomyces cerevisiae* cell wall stress induces the formation of these structures. This effect is dependent on multiple elements in the Cell Wall Integrity (CWI) MAPK signalling pathway, a signal transduction cascade responsible for the maintenance of cell integrity under adverse environmental conditions. Remarkably, P-body assembly requires the catalytic activity of the MAPK of the pathway, Slt2/Mpk1. In accordance with the control exerted by this signalling pathway, the timing of P-body formation is similar to that of the activation of the CWI pathway. Noticeably, mRNAs whose expression is regulated by this pathway localize in P-bodies after the cell is exposed to stress following a temporal pattern coincident with CWI pathway activation. Moreover, when these mRNAs are overexpressed in a mutant background unable to form visible P-bodies, the cells show hypersensitivity to agents that interfere with cell wall integrity, supporting that they play a role in the mRNA lifecycle under stress conditions.

## Introduction

In eukaryotic cells, post-transcriptional control of mRNA is an important mechanism for the regulation of gene expression. In this process, the specific localization and compartmentalization of mRNAs within the cytoplasm plays a key role^1^. The model yeast *Saccharomyces cerevisiae* has become an ideal system for studying these conserved cellular processes. In this context, a variety of cytoplasmic ribonucleoprotein (RNP) aggregates have been identified, the best characterized of which are processing bodies (P-bodies) and stress granules (SGs)^2–6^. It has been proposed that P-bodies contain translationally repressed mRNAs in combination with proteins involved in mRNA degradation, including subunits of the deadenylase CCR4/POP2/NOT complex, the decapping enzyme (Dcp1/Dcp2), the decapping activator Edc3 and the Lsm1-7 complex, the translation repressors and decapping activators Scd6, Dhh1 and Pat1, and the 5′-3′ exonuclease Xrn1 (for further details see^7^). Regarding the functions of P-bodies, these structures show an inverse relationship with translation, since trapping mRNA in polysomes due to the inhibition of translation elongation leads to the dissociation of P-bodies, in contrast to the stimulation of the assembly observed when the translation initiation is blocked^8^. These observations suggest that these foci participate in mRNA decay. However, yeast cells defective in P-body formation are not defective in basal control of translation repression and mRNA decay^9^. Moreover, recent data support a model in which P-bodies act as storage granules containing translationally repressed mRNAs and inactive decapping enzymes, while mRNA decay would take place throughout the cytoplasm^10^.

These cytoplasmic aggregates are highly dynamic, since in yeast cells grown in conditions of glucose starvation and subsequent refeeding, at least some mRNAs can leave P-bodies to reenter translation, being postulated as sites for transient mRNA storage^11,12^. In contrast, the SGs in yeast are considered aggregates of untranslating mRNAs in conjunction with certain translation initiation factors and other RNA binding proteins such as Pab1, Pub1 or Pbp1^4,5^. This explains why SGs are typically associated to stress conditions, which often involve a transient inhibition of translation initiation. Noticeably, in yeast, these granules are formed in a stress-dependent fashion^4,5,13,14^. In sum, several observations support the so-called mRNA cycle where cytoplasmic mRNAs cycle between polysomes, P-bodies and SGs^6,7^. This dynamic behaviour is favoured by the properties of liquid droplets exhibited by these structures^15^. P-body assembly is strongly induced in response to several stress conditions, such as glucose deprivation, osmotic, oxidative and DNA replication stress, heat or exposure to UV light, ethanol or NaN_3_^8,16,17^. This suggests that P-body aggregates would play a role under environmental stress conditions. Under hyperosmotic stress conditions, formation of P-bodies was substantially reduced in the short-term in yeast mutant strains lacking the mitogen-activated protein kinase (MAPK) of the High Osmolarity Glycerol MAPK pathway (HOG), Hog1^8,18^. Additionally, the Protein Kinase A (PKA) pathway, a key effector of glucose signalling in yeast, plays a general role in the regulation of P-body formation. In fact, constitutive PKA signalling inhibits P-body formation under a variety of stress conditions, and PKA activity inhibition is sufficient to induce P-body formation in non-stressed cells^17,19^. However, apart from these examples, the participation of signalling pathways associated to stress responses in the process of P-body assembly is largely uncharacterized.

The conservation of P-bodies from yeast to mammals suggests that they play important roles in the metabolism of eukaryotic mRNAs, especially under stress conditions. Remarkably, SGs and P-Bodies are closely associated with a variety of diseases, including neurodegenerative disorders^20^ and cancer^21^. Thus, information obtained from model organisms, such as yeast, is very useful when conducting mechanistic and functional analyses of the behaviour of these RNPs granules in higher organisms.

The Cell Wall Integrity (CWI) pathway is one of the MAPK pathways in yeast, being the main route responsible for maintaining cell wall homeostasis^22^. This pathway is very well conserved in the fungal kingdom^23^. When cell wall integrity is compromised, several cell membrane proteins (Mid2, Wsc1-3, and Mtl1) act as sensors of the damage and interact with the Guanine nucleotide Exchange Factor (GEF) Rom2, activating the small GTPase Rho1, which in turn activates the yeast protein kinase C (Pkc1). Pkc1 triggers the activity of a conserved MAPK module by phosphorylating the MAPKKK Bck1, which stimulates the redundant pair of MAPKKs Mkk1 and Mkk2, which finally phosphorylate and activate the MAPK Slt2/Mpk1. Ultimately, Slt2 induces damage-specific transcriptional responses mainly through the Rlm1 transcription factor^22,24^ in collaboration with the SWI/SNF and SAGA chromatin remodelling complexes^25,26^. Additionally, Slt2 has been involved in other Rlm1-independent functions as in the regulation of genes during G1/S transition of the mitotic cell cycle^27^, the regulation of the silencing function that couples cell growth and lifespan^28^ or the control of proteasoma abundance upon various stresses^29^.

Genome-wide transcriptional analysis of yeast mutant strains deleted in different elements of CWI have allowed the identification of differences in the regulatory mechanisms required for sensing the different stresses (for a review see^30^). For example, Congo red-mediated damage is sensed through the Mid2 sensor, whereas in the case of the enzymatic cocktail known as zymolyase damage is sensed through Hkr1, the sensor of the Sho1 branch of the high-osmolarity glycerol (HOG) pathway, and the effect of caspofungin, an inhibitor of β-1,3-glucan synthase, is sensed through Wsc1.

The CWI pathway is the key pathway for the regulation of the adaptive response that is elicited by the fungal cell under conditions that damage the cell wall, mainly inducing a transcriptional program to counterbalance cell wall stress situations^30^. However, little is known about the post-transcriptional regulatory events associated with this response.

In this work, we have demonstrated that when the yeast cell wall integrity is compromised, the formation of P-bodies is intensively promoted. Moreover, the assembly of these structures depends on signalling through the CWI pathway and, ultimately, on the kinase activity of the MAPK of this route. In addition, prototypic mRNAs whose expression is regulated by this route upon cell wall stress have been colocalized with P-bodies. In fact, the temporal pattern of formation of these structures, as well as the appearance of mRNA granules, mimics the activation of the CWI pathway under cell wall damage conditions. Additionally, we have shown that overexpression of some CWI-dependent mRNAs is toxic to P-body defective cells. Together, our results provide the first evidence that the response to cell wall damage not only activates a specific transcriptional program, but also regulates post-transcriptionally the cell wall-related mRNAs fate.

## Results and Discussion

### Cell wall stress induces P-body formation

**Figure 1:**
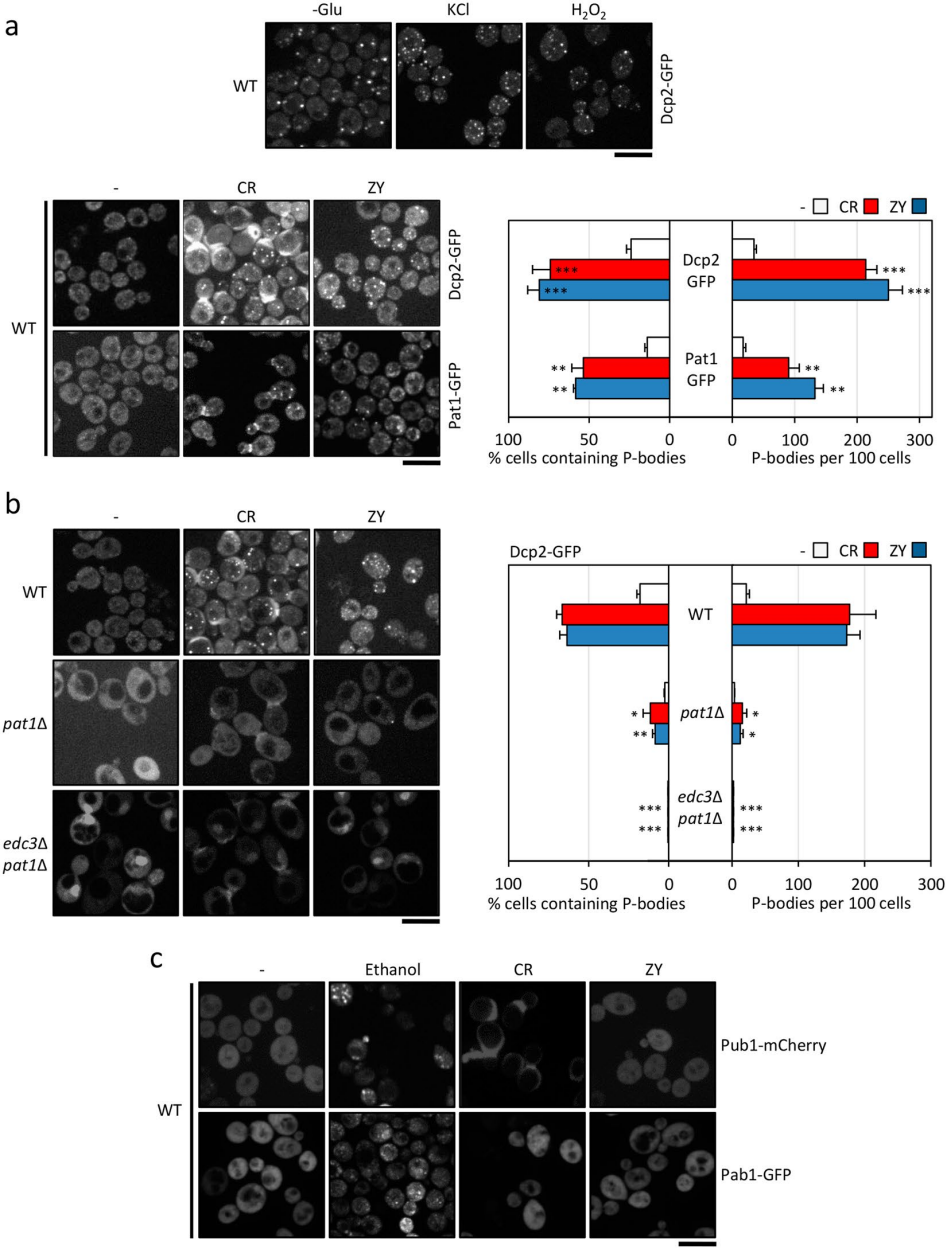
Formation of P-bodies is induced by cell wall stress. (**a**) Wild-type (WT) cells transformed with plasmids expressing a GFP-tagged version of Dcp2 or Pat1 growing in YPD were exposed to 30 µg/ml Congo red (CR) or 0.8 U/ml zymolyase (ZY) for one hour (lower panel) or 1 M KCl, 3 mM H_2_O_2_ and absence of glucose for 15 min (upper panel). P-body formation was then assessed using fluorescence microscopy. (**b**) P-body formation was studied using the Dcp2-GFP reporter in WT, *pat1*∆ and *edc3*∆ *pat1*∆ cells grown as indicated above. (**c**) Stress granule formation was not influenced by cell wall stress. WT cells expressing either Pub1-mCherry or Pab1-GFP were treated with CR and ZY as described in **a**, and with 15% ethanol for 30 minutes before being observed by fluorescence microscopy. The microscopy data are presented in the left panels and the quantitation of the results in the right panels. Non-treated cells are also included in each experiment as a control (−). The histograms show the number of P-bodies per 100 cells and the percentage of cells containing P-bodies. The data reflect the average and SD values obtained from three independent experiments (n > 100 cells). Statistical significance was determined using a two-tailed, unpaired, Student’s *t* test by comparing with no treatment conditions (**a**) or with the corresponding CR or ZY data from the wild-type strain (**b**) (*P ≤ 0.05, **P ≤ 0.01, ***P ≤ 0.001). Scale bar, 5 μm.

**Figure 2:**
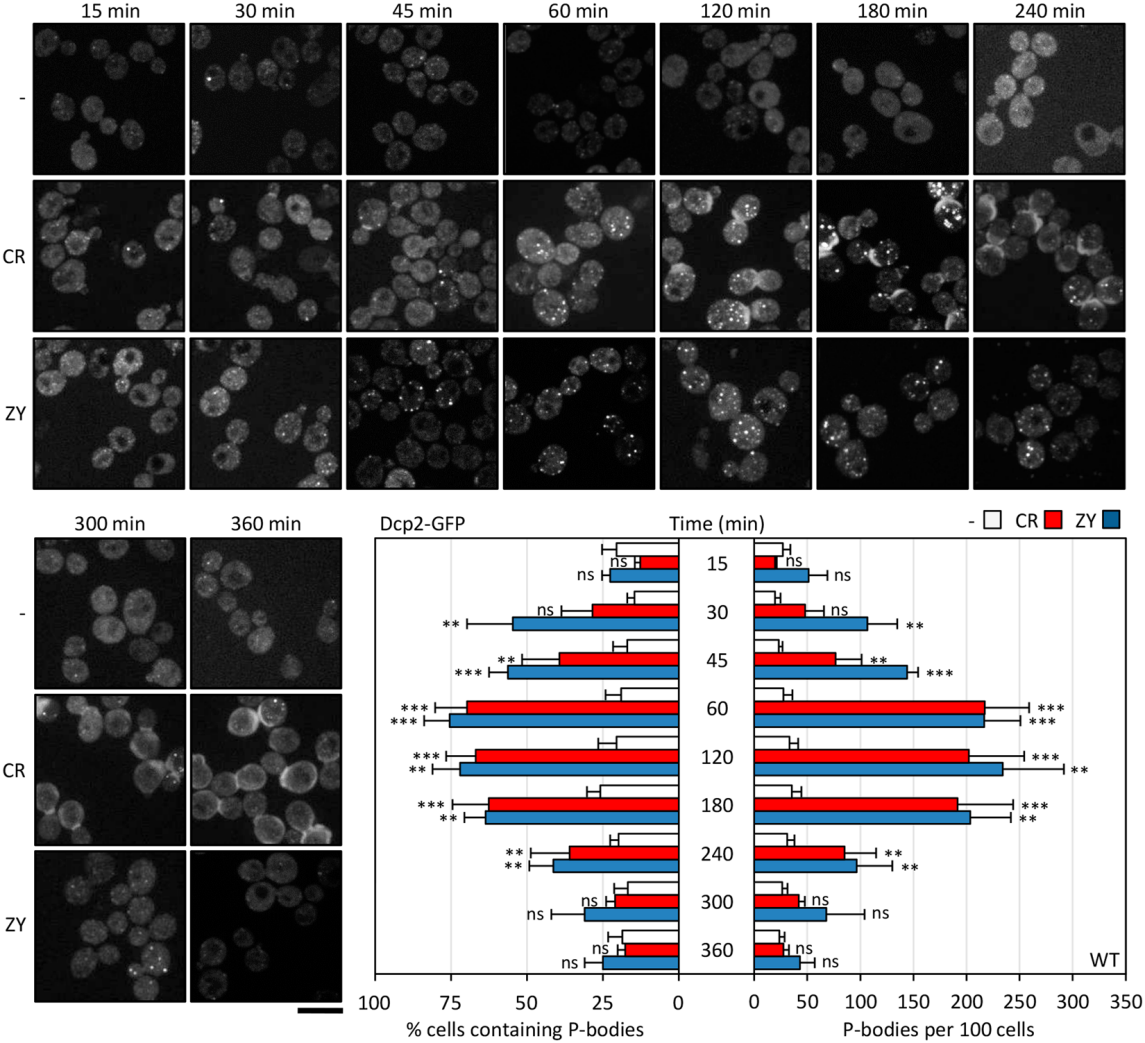
Time course of P-bodies formation in wild-type cells after cell wall stress. Wild-type (WT) cells expressing Dcp2-GFP as a P-body marker were treated with 30 µg/ml CR or 0.8 U/ml ZY and, in addition to the untreated control, were visualized by fluorescence microscopy at the times indicated. Scale bar, 5 μm. The histograms show the number of P-bodies per 100 cells and the percentage of cells containing P-bodies. The data reflect the average and SD values obtained from three independent experiments (n > 100 cells). Statistical significance was determined using a two-tailed, unpaired, Student’s *t* test by comparing with no treatment conditions for each time (*P ≤ 0.05, **P ≤ 0.01, ***P ≤ 0.001; ns, not significant).

**Figure 3:**
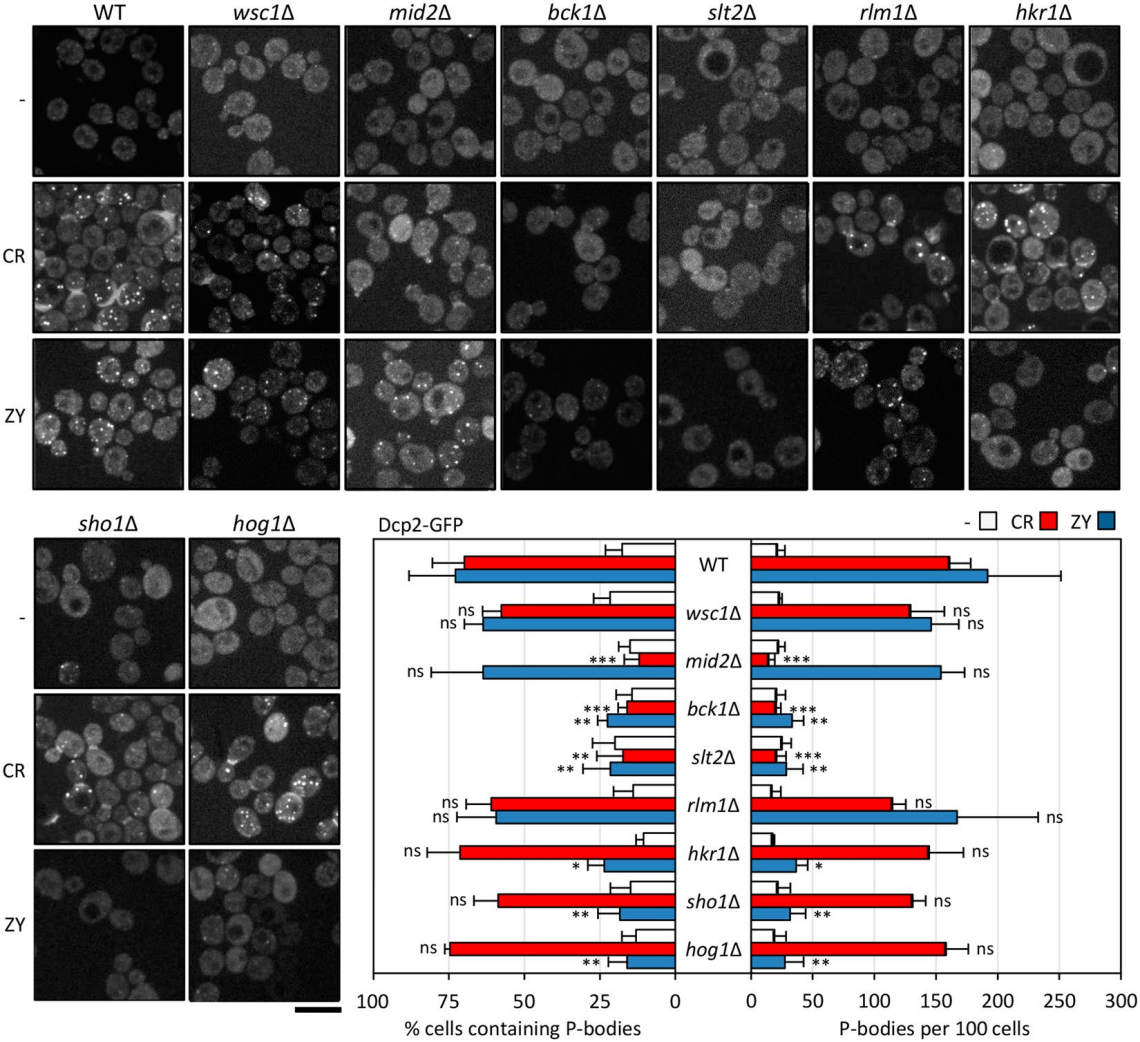
The CWI pathway controls P-body formation under cell wall stress. The wild-type strain (WT) and the indicated mutant strains transformed with the plasmid expressing Dcp2-GFP growing exponentially were treated with CR or ZY for one hour, as described in Fig. 1, and Dcp2-GFP containing granules were visualized by fluorescence microscopy. Quantitation of P-bodies from three independent experiments is included in the graph as described in Fig. 1. Statistical significance was determined using a two-tailed, unpaired, Student’s *t* test by comparing with the corresponding CR or ZY data from the wild-type strain (*P ≤ 0.05, **P ≤ 0.01, ***P ≤ 0.001; ns, not significant). Scale bar, 5 μm.

**Figure 4:**
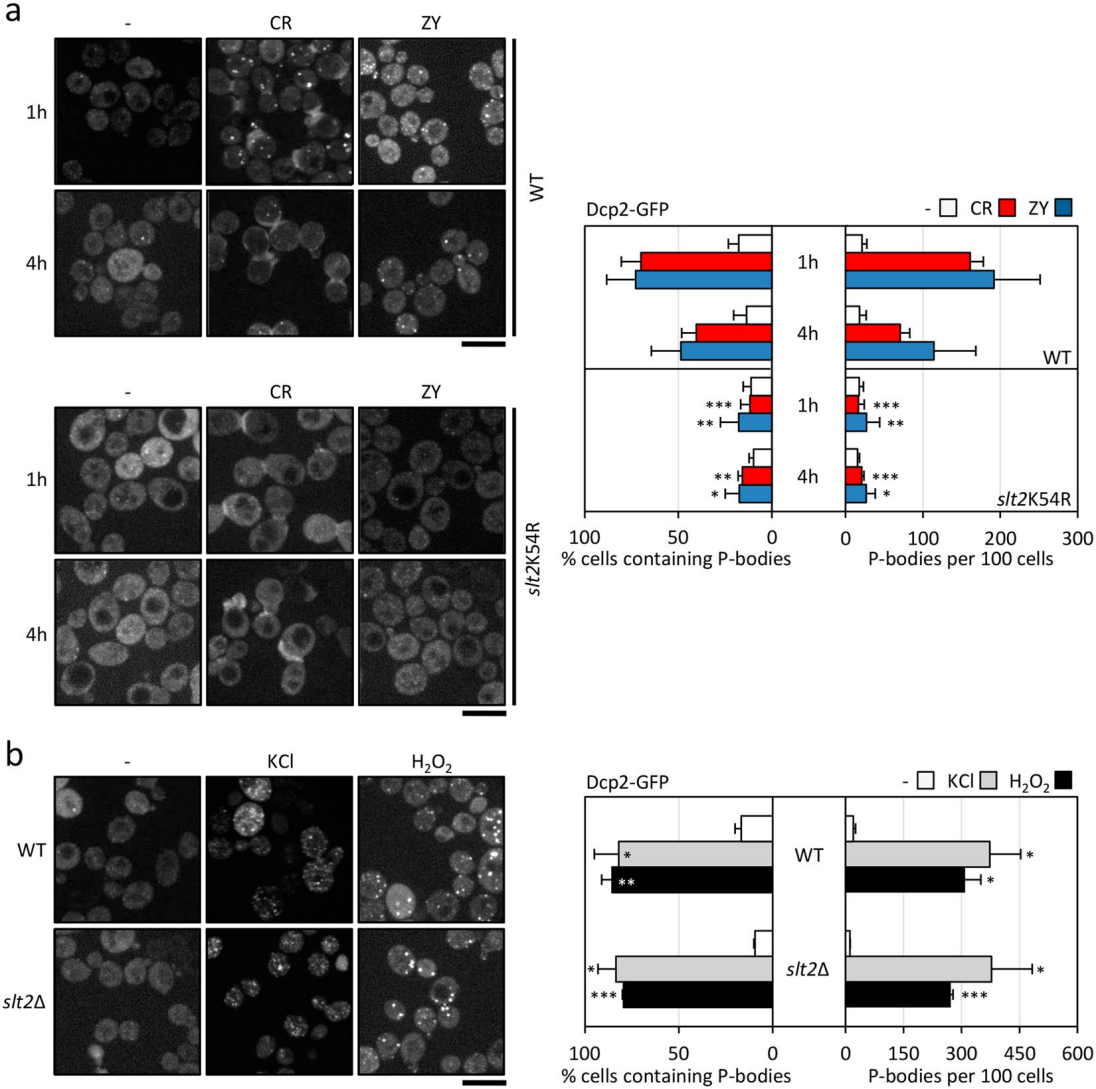
The activity of the MAPK Slt2 is essential for the increase in P-bodies under cell wall stress conditions. (**a**) Wild-type (WT) strain was transformed with the Dcp2-GFP plasmid. The *slt2*K54R strain corresponds to the *slt2*∆ mutant co-transformed with the Dcp2-GFP plasmid and a second plasmid expressing the kinase-death *slt2*K54R variant. Both strains were grown in the presence of 30 µg/ml CR or 0.8 U/ml ZY for one and four hours, and Dcp2-GFP foci were visualized by fluorescence microscopy. (**b**) P-bodies were visualized of in the WT and *slt2*∆ strains expressing Dcp2-GFP after growing in a medium containing 1 M KCl or 3 mM H_2_O_2_ for 15 min. The accompanying histograms reflect the quantitation of the microscopy data as described in Fig. 1. Statistical significance was determined using a two-tailed, unpaired, Student’s *t* test by comparing with the corresponding CR or ZY data from the wild-type strain (**a**) or no treatment conditions (**b**) (*P ≤ 0.05, **P ≤ 0.01, ***P ≤ 0.001). Scale bar, 5 μm.

**Figure 5:**
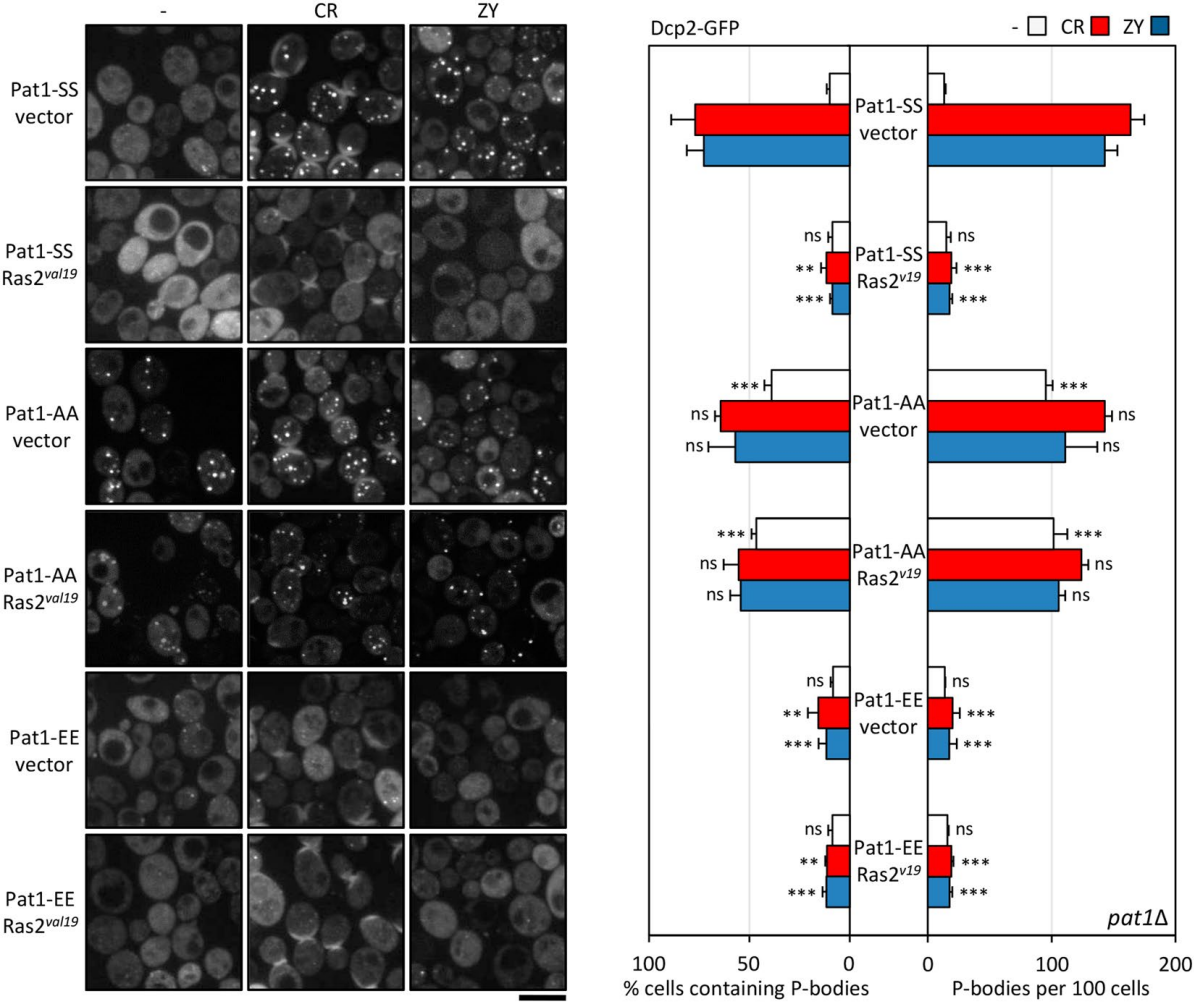
The presence of elevated PKA signaling activity inhibits P-body induction in response to cell wall stress. *pat1*Δ cells expressing the Dcp2-GFP protein and transformed with a plasmid that included the constitutively active Ras2^*val19*^ or the empty vector (vector) in combination with plasmids including the wild-type Pat1 (Pat1-SS), the non-phosphorylatable variant Pat1-AA or the phosphomimetic Pat1-EE variant were transferred to a medium containing 30 µg/ml CR or 0.8 U/ml ZY for one hour to induce P-body formation. Then, Dcp2-GFP foci were examined by fluorescence microscopy. Representative images are shown for both the control and treatment conditions. The quantitation of the microscopy data, from three independent experiments (n > 100 cells), is shown in the graphs. Statistical significance was determined using a two-tailed, unpaired, Student’s *t* test by comparing with the no treatment conditions, CR or ZY data from the *pat1*Δ strain expressing the Pat1-SS version (*P ≤ 0.05, **P ≤ 0.01, ***P ≤ 0.001; ns, not significant). Scale bar, 5 μm.

In order to investigate whether cell wall stress induces P-body foci formation, we primarily used GFP-tagged versions of two well-established reporters of P-body assembly: Dcp2 and Pat1. Wild-type cells were transformed individually with plasmids bearing these reporters and grown for one hour in the presence or absence of two compounds that interfere with cell wall integrity through different mechanisms of action. We used Congo red (CR), a dye that binds to chitin, and zymolyase (ZY), an enzymatic cocktail containing predominant β-1,3-glucanase activity, since the transcriptional responses elicited by exposure to these compounds have been extensively characterized in yeast^31–33^. The microscopic observation of Dcp2-GFP and Pat1-GFP expressing cells showed that cell wall stress strongly induced the assembly of P-bodies, both in terms of the number of cells in which P-bodies were observed and the number of foci per cell (Fig. 1a). As expected, this effect was also observed in cells expressing Dcp2-GFP after their exposition to other stress conditions (glucose starvation and presence of KCl or H_2_O_2_) previously associated to P-body formation (Fig. 1a upper panel). To further confirm the association between cell wall stress and P-body formation, the same experiments were performed using the *pat1*Δ mutant and the *edc3*Δ *pat1*Δ double mutant, previously described as defective in P-body formation^5,19^. As shown in Fig. 1b, Dcp2-GFP-containing granules after cell wall stress were drastically reduced in the *pat1*Δ strain and completely absent in the *edc3*Δ *pat1*Δ mutant. It is important to note that in the microscopy images showing CR treated cells (Figs 1–5), many cells show some fluorescence signal at the mother-bud neck. This signal exhibits a localization pattern different to that corresponding to P-bodies and it is a consequence of the CR fluorescence^34^. Although CR emission is detected in the red channel, after prolonged incubation of the cells with CR, such as the conditions used in this work, a strong accumulation of the dye occurs at the surface regions with higher chitin content (CR binds to this cell wall polymer), like the septum between the mother and daughter cells. This signal is weakly detected in the green channel used to visualize GFP.

Since the assembly of SGs (another type on RNP granule) has been associated with particular stress conditions, we investigated whether SGs were formed under cell wall stress conditions. To achieve this, we monitored the intracellular localization of two components of SGs fused to fluorescent proteins, Pub1-mCherry and Pab1-GFP. Contrary to the P-body experiment, we only detected a diffuse fluorescence signal distributed throughout the cytoplasm, which never concentrated in discrete foci (Fig. 1c). However, the same cells grown in the presence of 15% ethanol for 30 minutes efficiently formed SGs, as previously reported^17^(Fig. 1c).

In contrast to many other environmental stresses that activate rapid cellular responses, cell wall stress triggers in yeast a more delayed adaptive response after exposure. Thus, we monitored P-body formation at different times during a time-course experiment (15, 30, 45, 60, 120, 180, 240, 300 and 360 minutes) after CR or ZY treatment in wild-type cells expressing Dcp2-GFP. As shown in Fig. 2, the P-body formation profile was very similar in response to both cell wall damaging agents. After 15–30 minutes of treatment, Dcp2-GFP-containing foci started to increase in comparison with non-treated cells, reaching a peak after one to three hours of exposure. After this period, the percentage of cells showing detectable P-bodies, as well as the total number of foci, diminished progressively to values comparable to those observed in unstressed cells (Fig. 2). Notably, the timing of P-body formation was similar to the global profile of Slt2 phosphorylation, the MAPK of the CWI pathway, in response to cell wall stress, previously described^31,35^. It is important to remark that our results support the existence of a very fine temporal regulation of the formation of P-bodies through different signalling pathways, depending on each type of stress and the specific cellular requirements under these conditions. In fact, the absence of glucose causes the assembly of these structures in 10 min^11^, and similar values have been described for osmotic and UV^8^ or oxidative^17^ stress, in contrast to the slower response described here for cell wall damage.

Taking these results together, we conclude that cell wall stress induces *bona fide* P-body assembly following a temporary profile that mimics that of the activation of the CWI pathway.

### P-body assembly under cell wall stress is dependent on the activation of the CWI pathway

To characterize the participation of the CWI pathway in P-body assembly, we monitored Dcp2-GFP localization in the absence or presence of CR for one hour, in several mutant strains lacking key elements of this signalling pathway, namely the sensors Wsc1 and Mid2, the MAPKKK Bck1, the MAPK Slt2 and the transcription factor Rlm1. As shown in Fig. 3, the increase in visible P-body formation was completely blocked in mutants lacking *MID2* or the *SLT2* and *BCK1* elements of the CWI pathway MAPK module, where activation of Slt2 is blocked. These results highlight the importance of the activation of the CWI pathway in the formation of P-bodies under cell wall stress conditions. In fact, in strains lacking elements not required for CR-induced Slt2 activation (phosphorylation), such as the *WSC1* sensor, the formation of these structures was unaffected. Interestingly, as deduced from the correct formation of P-bodies in an *rlm1∆* strain, the transcription factor Rlm1 that controls the expression of the majority of the genes induced in response to stress^30^ is not involved in this process. To further analyse the participation of the CWI pathway in P-body formation, we performed the same experiments using an alternative stimulus, growing the yeast cells in the presence of zymolyase. Under these stress conditions, Slt2 activation requires the participation of the Sho1 branch of the HOG MAPK pathway, in particular the transmembrane protein Sho1 and the Hkr1 sensor, but not the Mid2 or Wsc1 sensors of the CWI^35,36^. In these experiments, we evaluated the effect on P-body formation of yeast mutants deleted in these elements during zymolyase exposure. As shown in Fig. 3, the absence of Sho1, Hkr1 or Hog1, in addition to the absence of Bck1 or Slt2, prevented P-body formation, whereas Mid2, Wsc1 or Rlm1 did not play a prominent role. Conversely, in strains lacking Hkr1, Sho1 or Hog1, P-body assembly efficiency after CR treatment was similar to that observed in the wild-type strain.

As further proof of the essentiality of Slt2 activity in P-body generation, we took advantage of the K54R mutant form of the MAPK Slt2, consisting of a mutation within the ATP-binding site, which blocks the catalytic activity of the protein^37^. This allele, borne on a centromeric plasmid, was unable to restore Dcp2-GFP foci formation in the *slt2*Δ strain, either in the presence of CR or ZY for one hour (Fig. 4a). This effect was also observed even after longer treatment times (up to four hours), supporting the notion that Slt2 activity is necessary not only for the initial assembly of these structures, but also for their maintenance over time (Fig. 4a). To test whether Slt2 is specifically involved in P-body formation after cell wall stress, we monitored Dcp2-GFP localization during hyperosmotic (1 M KCl) and oxidative stress (3 mM H_2_O_2_), two types of stress previously associated with these structures^17^. The number of cells bearing Dcp2-GFP granules, as well as the percentage of cells presenting them, was not altered in the *slt2*∆ mutant when compared to the values obtained in the wild-type strain (Fig. 4b). These results support the argument that Slt2 plays a cell wall damage-specific role in this process.

In sum, these results emphasize that both Slt2 activation due to the sensing of specific cell wall injuries, and the corresponding kinase activity of the phosphorylated Slt2 are essential for P-body assembly after cell wall stress. This regulatory mechanism is identical to that exerted by Slt2 on the adaptive transcriptional response triggered under these conditions. Remarkably, the transcriptional response regulated by Rlm1 is not critical for this phenomenon, indicating that alternative substrate/s of Slt2 are involved. In this context, our laboratory is currently focusing its efforts on identifying the target/s on which the MAPK Slt2 acts to control the formation of these mRNA-protein aggregates, considering that post-translational modifications of P-body proteins, e.g. phosphorylation events, have been proposed to modulate P-body formation^10^. Moreover, the fact that in human cells is also present a MAPK ortholog of the yeast Slt2, ERK5^38^, which is regulated by diverse mitogens and stresses, makes it possible to hypothesise a participation of the ERK5 pathway in the formation of these assemblies in human cells.

### P-body formation induced by cell wall damage is blocked by constitutive PKA signalling

Although little is known about the signalling pathways that mediate P-body assembly, previous studies^17,19^ have demonstrated that the cAMP-dependent protein kinase (PKA), responsible for coordinating cell growth with nutrient availability, impacts on this process, at least through the modulation of the phosphorylation status of Pat1, which is one of its targets. In fact, P-body formation as a consequence of a variety of stress conditions is abrogated in the presence of constitutive PKA signalling^17^. Regarding cell wall stress, we found that the number of P-bodies per cell observed in a yeast strain bearing the wild-type version of Pat1 (Pat1-SS) under CR or ZY treatment was significantly diminished in the presence of elevated PKA activity expressing a dominant active allele of the small GTP-binding protein Ras2 (Ras2^*val19*^), which positively regulates PKA activity (Fig. 5).

Moreover, we tested whether Pat1 phosphorylation mediated by Ras2-PKA activation was required for the observed P-body inhibition. To achieve this, Pat1-AA or Pat1-EE variants were expressed in the *pat1*∆ strain expressing Dcp2-GFP and bearing Ras2^*val19*^ or the corresponding empty vector. The Pat1-AA protein has alanine residues replacing the two serines that are phosphorylated by PKA, Ser-456 and Ser-457^19^, thus consisting of a non-phosphorylatable version, while in the Pat1-EE protein, the presence of a glutamic acid can functionally substitute a phosphorylated serine, and thus this variant mimics the PKA phosphorylated form of Pat1. We found that the yeast strain expressing Pat1-AA formed P-bodies constitutively (Fig. 5), in agreement with the previous observation that the inactivation of PKA signalling is sufficient for P-body formation in the absence of stress^19^. Moreover, the co-expression of Ras2^*val19*^ was not able to block P-body formation neither in the absence nor in the presence of stress (Fig. 5). Contrary, the recruitment of Dcp2 to cytoplasmic foci in the presence of CR or ZY was diminished in cells containing the Pat1-EE variant (Fig. 5), which agrees with the effect observed above in wild-type cells (Pat1-SS) carrying the Ras2^*val19*^ allele. These results indicate that, as previously reported for other stress conditions^17^, in the case of cell wall stress, PKA inhibits P-body foci formation through Pat1 phosphorylation. From all the available data it is possible to postulate two possible mechanisms in relation to the global control exerted by PKA on the formation of P-bodies induced by multiple unrelated stresses. One where the stress itself results in an inhibition of the PKA activity, as in conditions of absence of glucose, therefore inducing the assembly of P-bodies, and a second one, where a given stress type does not affect PKA activity, although the ectopic activation of PKA interferes with P-body assembly upon that condition. Concerning the cell wall stress caused by CR or ZY, the first option is unlikely because in large-scale transcriptional studies carried out with these agents no transcriptional effects typically associated to the modulation of the PKA activity were detected^31,32^.

### CWI-regulated mRNAs are localized to P-bodies under cell wall stress conditions

**Figure 6:**
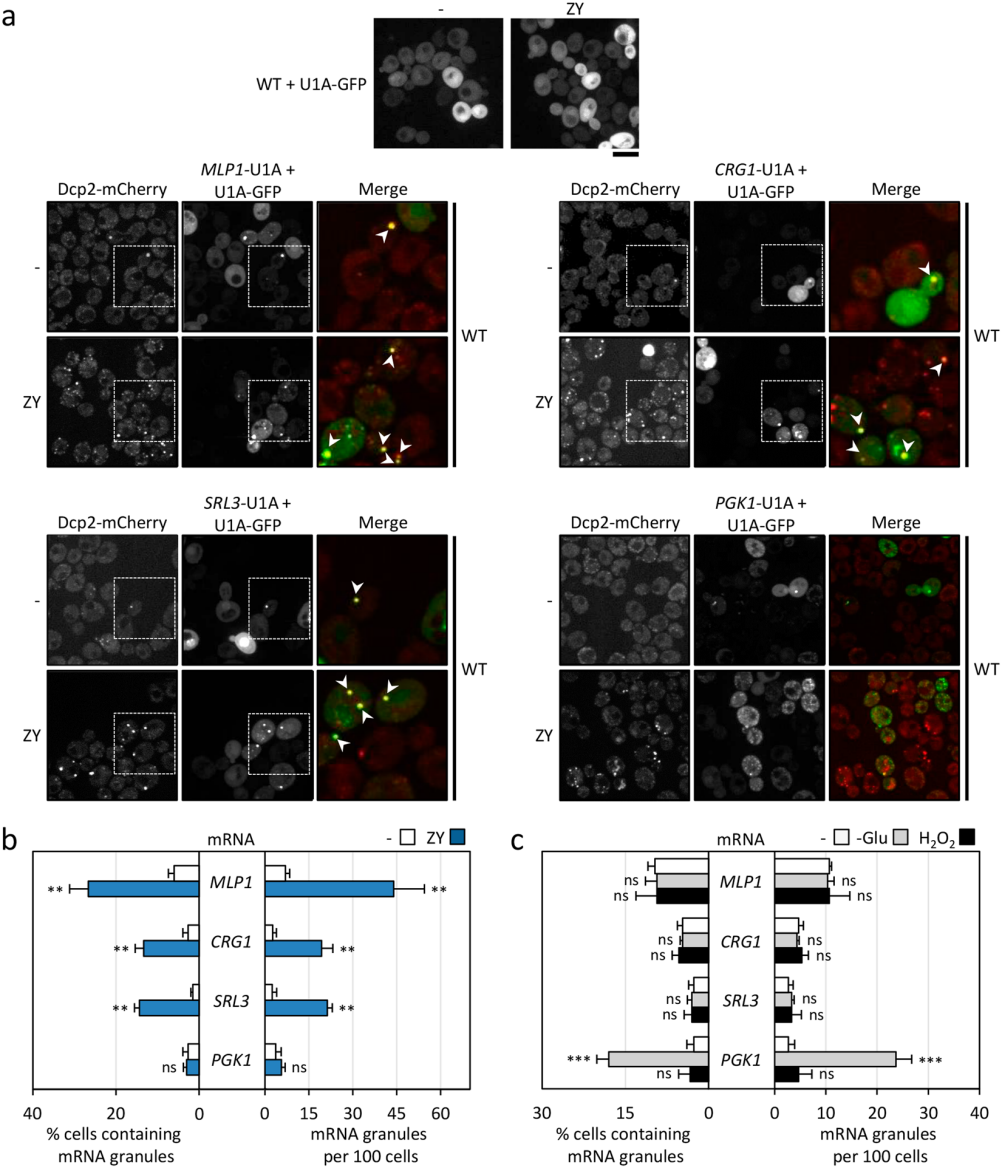
Cell wall stress induces localization of CWI-responsive mRNAs to P-bodies. (**a**) Confocal microscopy images of wild-type (WT) cells containing Dcp2-mCherry, coexpressing *MLP1*/*CRG1*/*SRL3* or *PGK1-*U1A mRNA and U1A-GFP protein to allow analysis of mRNA localization growing in the absence or presence of 0.8 U/ml ZY for two hours, are represented. Control cells expressing only U1A-GFP protein in the absence of target mRNA are shown (upper panel). The colored overlay images show a 4.4 times enlargement of the image sections included in the squares and depict examples where the mRNAs colocalize with P-bodies (white arrowheads). (**b**) The graphs display the quantitation of the mRNA granules shown in (**a**) from three independent experiments (n > 100 cells). (**c**) Quantification of mRNA granules (GFP foci) in strains described in (**a**), treated with 3 mM H_2_O_2_ or starved for glucose for 15 min, is shown in the graphs. Statistical significance was determined using a two-tailed, unpaired, Student’s *t* test by comparing with no treatment conditions (*P ≤ 0.05, **P ≤ 0.01, ***P ≤ 0.001; ns, not significant).

It is well known that the CWI pathway mediates the transcriptional response necessary for maintaining cellular viability under situations where cell wall integrity is jeopardized^30^. Since we had demonstrated the relation between the activation of the CWI pathway and the formation of P-bodies, we next investigated whether the mRNAs of genes regulated by this pathway localized to P-bodies under cell wall stress conditions. To achieve this, we visualized *in vivo* the localization of *MLP1/KDX1*, *CRG1* and *SRL3* mRNAs using the U1A-based tagged RNA system^39^. These mRNAs were selected because their expression levels are significantly increased under cell wall stress in a CWI-dependent manner^31,32^. To monitor the cellular distribution of these mRNAs, the wild-type strain expressing a genomically mCherry-tagged Dcp2 was transformed with two plasmids. One plasmid contained the promoter and ORF of the transcript of interest followed by a module including multiple binding sites for the U1A human protein, inserted after the translation termination codon. Simultaneously, the U1A-GFP fusion protein was expressed from a second plasmid. The three mRNAs under study were distributed diffusely throughout the cytoplasm during growth in the absence of stress (Fig. 6a). However, after two hours of zymolyase treatment, strains co-expressing the *MLP1*, *CRG1* or *SRL3* mRNA containing U1A-binding sites and the U1A-GFP fusion protein showed a clear concentration of the GFP signal in cytoplasmic foci, which colocalized in more than a 90% with Dcp2-mCherry containing assemblies (Fig. 6a,b). Moreover, there are Dcp2-containing foci in which mRNA fluorescent signal is not detected. This could be due to the presence of mRNA amounts below the detection level and/or the existence of different subpopulations of P-bodies bearing specific mRNAs or even lacking them. As expected, control strains expressing the U1A-GFP without the target mRNA did not show any concentration of the GFP signal in response to the presence of stress (Fig. 6a). Interestingly, when we visualized the mRNA of the *PGK1* gene, encoding the glycolytic enzyme phosphoglycerate kinase, which is not regulated by the CWI pathway, the number of mRNA granules was not increased in the presence of ZY and these granules were only modestly detected in visible P-bodies (Fig. 6a,b). This supports the notion that specific mRNAs induced during cell wall stress are accumulated in P-bodies. This finding is reinforced by the fact that when cells were treated with other unrelated stresses, such as the oxidative stress caused by hydrogen peroxide or glucose deprivation, no induction of *MLP1*, *CRG1* or *SRL3* mRNA granules was observed (Fig. 6c). However, as described^8^, the number of granules containing *PGK1* mRNA was augmented specifically under conditions of glucose deprivation (Fig. 6c), colocalising with P-bodies.

**Figure 7:**
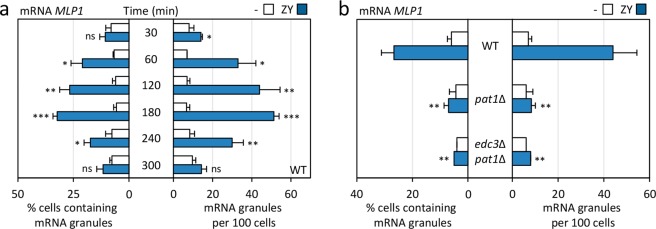
The induction of mRNA granules under cell wall stress conditions is associated with P-body formation. (**a**) Time course analysis of the appearance of *MLP1-*U1A mRNA granules in wild-type (WT) cells coexpressing *MLP1-*U1A mRNA and U1A-GFP protein after ZY exposure (0.8 U/ml) at the indicated times are shown in the graphs. The data reflect the average of mRNA granules counts from three independent experiments analysed by fluorescence microscopy. (**b**) Following the strategy described above, the presence of *MLP1-*U1A mRNA granules resulting from cell wall stress (two hours of ZY treatment) was quantified in WT and yeast strains defective in P-body formation (*pat1*∆ and *edc*3∆ *pat1*∆). Statistical significance was determined using a two-tailed, unpaired, Student’s *t* test by comparing with no treatment conditions for each time (**a**) or ZY data from the wild-type strain (*P ≤ 0.05, **P ≤ 0.01, ***P ≤ 0.001; ns, not significant).

To further investigate the kinetics of mRNA localization in P-bodies, we monitored the localization of *MLP1* mRNA in a time-course experiment during five hours of zymolyase treatment. As shown in Fig. 7a, *MLP1-*tagged RNA recruitment was slightly delayed in comparison to P-body assembly (Fig. 2), reaching a peak after three hours of treatment. Interestingly, the formation of *MLP1* mRNA-containing granules was severely affected in strains deficient in P-body assembly, such as *pat1*∆ or *edc3*∆ *pat1*∆ (Fig. 7b).

These results suggest that P-body assembly should be advantageous for cellular fitness under cell wall stress conditions. One possibility is that the localization of specific mRNAs in these structures could be required to optimize the global mRNA lifecycle, perhaps in concert with translational regulation.

**Figure 8:**
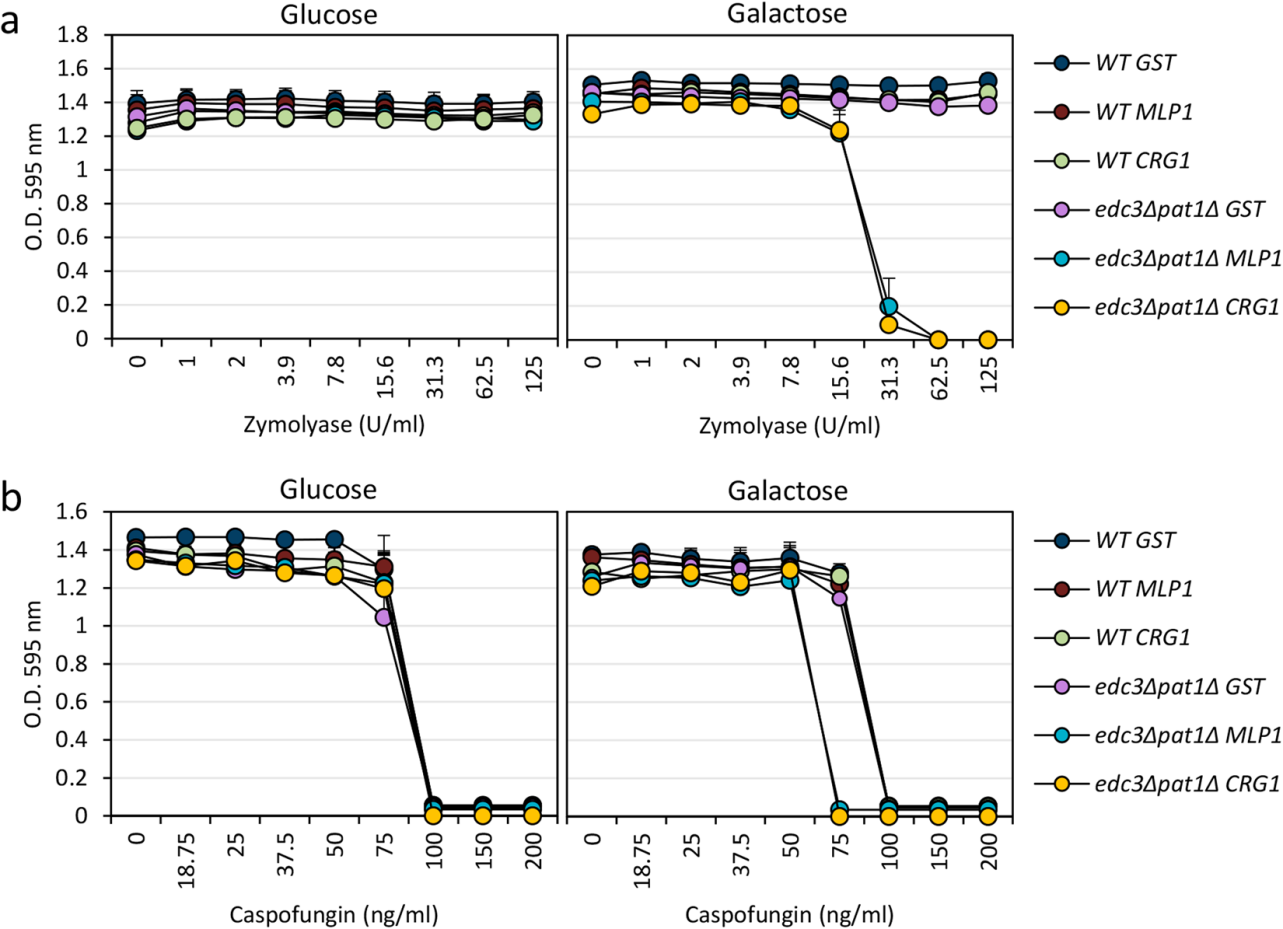
A high level of expression of CWI-related genes affects the viability of mutants unable to form P-bodies. Sensitivity assays to zymolyase (**a**) or caspofungin (**b**) of the wild-type (WT) and *edc3*∆ *pat1*Δ strains containing the *GAL1/10pr-GST*, *GAL1/10pr-GST-MLP1* or *GAL1/10pr-GST-CRG1* plasmids were performed and measured in media containing galactose (YPSG, induction of gene expression from *GAL* promoter) or glucose (YPD, repression of gene expression) as described in *Methods*. The graphs depict the average and SD values obtained from three independent experiments.

In this regard, previous research has found that deregulation of gene expression of specific genes whose transcripts localized to P-bodies is deleterious for cell growth of P-body mutant strains^40,41^. To investigate this in our stress conditions, we evaluated the effect of the overexpression of versions of *MLP1* and *CRG1* fused to GST on the cellular growth of the wild-type strain and a P-body deficient mutant (*edc3*∆ *pat1*∆) in the absence or presence of cell wall stress. In experiments to determine minimum inhibitory concentrations using a microdilution method, we observed that expression of both genes from the *GAL* promoter in the *edc3*∆ *pat1*∆ background was highly toxic in the presence of zymolyase (Fig. 8a). Moreover, this effect was also observed, although to a lesser degree, when caspofungin was used, which inhibits β-1,3-glucan synthesis (Fig. 8b). Remarkably, expression of GST did not have any effect on the levels of growth of either strain, showing a behaviour similar to that observed when experiments were carried out in the YPD medium (absence of overexpression). An additional observation from these experiments was that the *edc3*∆ *pat1*∆ strain was able to withstand the presence of zymolyase or caspofungin at the same level of the wild-type strain (see graphs corresponding to growth in glucose-containing medium in Fig. 8). This phenomenon could be explained by a recent observation, using the nanoparticle tracking technology^42^, that in the absence of different proteins constituent of visible P-bodies by fluorescence microscopy, other types of protein-protein and/or protein-RNA interactions are stablished allowing the assembly of non-visible P-body like particles that could functionally replace them.

Taken together, results from this work support the notion that the CWI pathway is responsible for the fine tuning of the formation of PBs in situations where the cell wall integrity is compromised, in coordination with expression regulation of mRNAs dependent on this signalling pathway that are located in these structures. Ongoing efforts in our laboratory aim to decipher what is the contribution of P-body formation to the cellular physiology upon cell wall stress conditions. Finally, we expect that the information provided by this work can be useful to characterise P-body formation under cell wall stress in other pathogenic fungi of clinical interest.

## Methods

### Yeast strains and plasmids

Experiments were performed with the *Saccharomyces cerevisiae* BY4741 strain (*MAT***a**; *his3*Δ1; *leu2*Δ0; *met15*Δ0; *ura3*Δ0) and mutant derivatives provided by Euroscarf (Frankfurt, Germany). The yeast strains from this collection used in this work were: *wsc1*Δ, *mid2*Δ, *bck1*Δ, *slt2*Δ, *rlm*1Δ, *hkr1*Δ, *sho1*Δ, *hog1*Δ and *pat1*Δ. To generate the *edc3*Δ *pat1*Δ double mutant strain, the *EDC3* gene was replaced by the *HIS3MX6* module in the *pat1*Δ strain using the SFH PCR-based method, as previously described^43^. The tagged strain *DCP2-mCherry::HIS3* was obtained using the one-step polymerase chain reaction (PCR)-mediated technique for gene modification^44^. Moreover, mCherry was amplified using PCR from a variant of the pBS34 plasmid (provided by Eric Muller, [Addgene plasmid #83796])^45^, in which the *KanR* selection marker has been replaced by the *HIS3MX6*. The resulting fragment was integrated by homologous recombination into the *DCP2* locus in the wild-type BY4741 background. Correct integration was confirmed using a PCR-based strategy.

Plasmids expressing the protein fusions Dcp2-GFP (pRP1315), Pat1-GFP (pRP1501), Pub1-mCherry (pRP1661), Pab1-GFP (pRP1362), Pgk1-U1A (pRP1354) and U1A-GFP (pRP1194) under the control of the native promoters, all of which bearing *URA3* as a selection marker except U1A-GFP (*LEU2* marker), were kindly provided by Dr. Roy Parker (Department of Chemistry and Biochemistry, University of Colorado, Boulder, CO, USA)^46^. Plasmids including the Pat1 variants Pat1-SS (wild-type protein including Ser-456 and Ser-457 phosphorylated by PKA), Pat1-EE (Pat1 variant where both of the aforementioned serines are replaced by a glutamic acid) and Pat1-AA (with both serines replaced by alanine), cloned in the pRS413 vector, were provided by Paul K. Herman (Department of Molecular Genetics, The Ohio State University, Columbus, OH, USA)^19^. Plasmid pTS120 expressing a constitutively active Ras2 (*RAS2*^*val19*^ allele) was provided by Dr. Michael N. Hall (Division of Biochemistry, Biozentrum, University of Basel, Switzerland)^47^. Plasmid pRS315-*slt2*K54R (p2193)^37^, was provided by David E. Levin (Department of Molecular and Cell Biology, Boston University School of Medicine, Boston, MA, USA). To construct Mlp1-U1A, Crg1-U1A and Srl3-U1A plasmids, the *PGK1* Promoter-ORF and 3′UTR regions from the pRP1354 plasmid were replaced with those of *MLP1*, *CRG1* and *SRL3* present in the *Xho*I/*Bam*HI and *Spe*I/*Not*I fragments, respectively, obtained by PCR from genomic DNA using primers containing the indicated restriction sites. The fragment sizes of the promoter-ORF regions were 2280 bp, 1854 bp and 1287 bp for *MLP1*, *CRG1* and *SRL3*, respectively. In the case of the 3′UTRs regions, the fragment sizes were 345 bp, 375 bp and 351 bp, respectively.

Plasmids expressing fusions of *MLP1* and *CRG1* to GST, in addition to the plasmid control expressing only GST, under the control of the *GAL1/10* promoter, were obtained from the collection of Yeast GST-tagged ORFs (Dharmacon/Open Biosystems, Lafayette, CO, USA).

### Growth conditions

Routinely, yeast cells were grown overnight at 24 °C in liquid SD medium (0.17% yeast nitrogen base, 0.5% ammonium sulphate, 2% glucose, supplemented with the required amino acids) for strains transformed with plasmids or YPD (1% yeast extract, 2% peptone and 2% glucose) to an optical density of 0.8–1 at 600 nm. Next, the culture was refreshed in YPD to an optical density of 0.1 at 600 nm, grown for 2.5 hours, and then divided into two parts. One part, the non-treated culture, continued growing under the same conditions, while the other one was supplemented when required with sublethal concentration of Congo red (30 µg*/*ml; Merck KGaA, Darmstadt, Germany), zymolyase from *Arthrobacter luteus* (0.8 U/ml; MP Biomedicals, CA, USA), KCl (1 M; PanReac AppliChem, Castellar del Vallès, Barcelona, Spain) or H_2_O_2_ (3 mM; PanReac AppliChem, Castellar del Vallès, Barcelona, Spain). Finally, cells were collected at the indicated times for each assay as described below. In the case of phenotypic analyses, yeast cells were grown overnight at 24 °C in liquid YPS medium (1% yeast extract, 2% peptone and 0.5% sucrose) to an optical density of 0.8–1 at 600 nm. Next, the culture was refreshed in YPS to an optical density of 0.1 at 600 nm, grown for 2.5 hours and then divided into two parts. One part continued growing under the same conditions (non-expressing conditions), while the other was supplemented with galactose (2% final concentration, YPSG medium) and both were incubated again for two hours. The caspofungin used in sensitivity tests was kindly provided by Merck Sharp and Dohme [MSD] Research Laboratories (Kenilworth, NJ, USA).

### Microscopy

Exponentially growing yeast cells were quickly collected by centrifugation at 5.000 rpm for 30 seconds. Then, the culture medium was decanted, and cell pellets were resuspended in the volume of medium that remained and immediately observed under a confocal fluorescence microscope. Images were taken using a motorized Olympus IX81 microscope (Olympus Corporation, Tokyo, Japan) equipped with a Yokogawa Spinning Disk confocal system (Roper Technologies, Sarasota, FL, USA), and an EMCCD Evolve camera (Photometrics, Tucson, AZ, USA) using MetaMorph software (Molecular Devices, San Jose, CA, USA). Images were analysed using the Fiji-ImageJ software^48^. All images were generated from maximum-intensity projections of 3–5 Z-sections spaced at 0.3–0.5 µm. To count P-body foci, Fiji-ImageJ “thresholding” and “analyze particle” functions were used, as described by Buchan *et al*.^46^. P-body foci counting was performed on a minimum of 100 cells from at least three independent experiments.

### Phenotypic analyses

To determine the sensitivity of yeast strains to zymolyase and caspofungin, a microdilution method was carried out. These assays were done in 96-well microtiter plates, with serial dilutions of zymolyase 20 T (from 125 to 1 U/ml) or caspofungin (from 200 to 18.75 ng/ml) prepared in a final volume of 150 µl of YPD (for cells grown in YPS) or YPSG medium (for cells grown in the same medium). Each well was inoculated with ~10^4^ cells from an exponentially growing culture. Plates were incubated for 72 hours at 30 °C, and cell growth was determined by measuring absorbance at 595 nm on an ELISA microplate reader.
