# Author Correction: Signalling through the yeast MAPK Cell Wall Integrity pathway controls P-body assembly upon cell wall stress

**DOI:** 10.1038/s41598-019-52664-x

**Published:** 2019-11-07

**Authors:** Raúl García, Verónica Pulido, Sara Orellana-Muñoz, César Nombela, Carlos R. Vázquez de Aldana, José M. Rodríguez-Peña, Javier Arroyo

**Affiliations:** 1 Departamento de Microbiología y Parasitología, Facultad de Farmacia, Universidad Complutense de Madrid, IRYCIS, Madrid, 28040 Spain; 2 Instituto de Biología Funcional y Genómica, IBFG-CSIC. Universidad de Salamanca, Salamanca, 37007 Spain

Correction to: *Scientific Reports* 10.1038/s41598-019-40112-9, published online 28 February 2019

The Article contains an error in Figure 4a in which an incorrect image is shown for the yeast strain slt2K54R treated with ZY for 4 h. The correct Figure 4 appears below.

**Figure 4:**
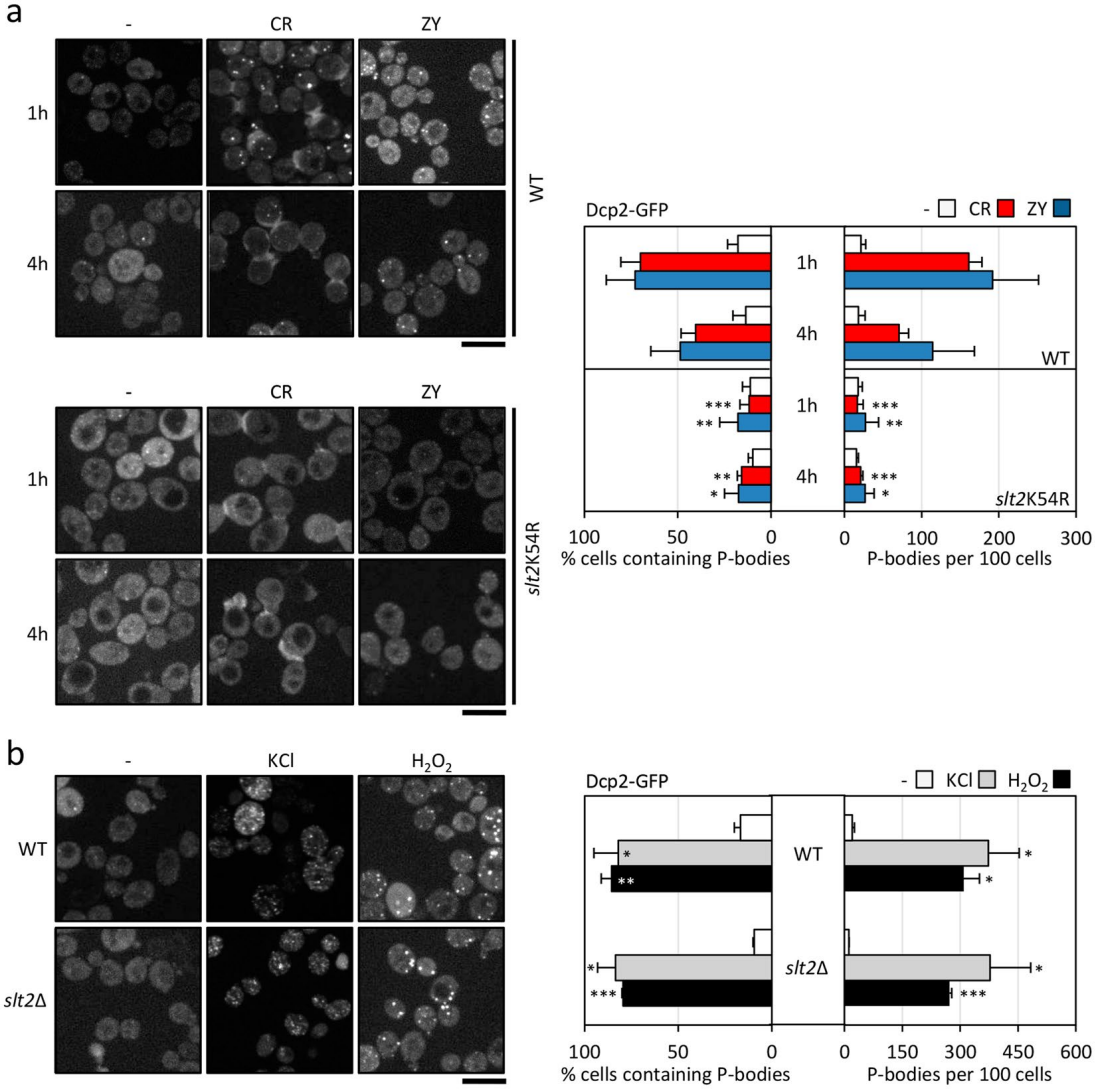
.

The conclusions of the Article are unaffected by these changes.

